# Supplementary material for: Chronic Hepatitis B Viral Activity Enough to Take Antiviral Drug Could Predict the Survival Rate in Malignant Lymphoma
Source: Viruses. 2022 Aug 31;14(9):1943. doi: 10.3390/v14091943 (PMC9500776; doi:10.3390/v14091943)
Supplement: Supplementary file 1 [file viruses-14-01943-s001.zip › Viruses Supplement figure.pdf]

Supplement figure legends

Supplement Figure 1. Study flow of chronic hepatitis B patients with and without malignant lymphoma from 2002 to 2016.

Supplement Figure 2. Newly diagnosed malignant lymphoma incidence in treatment-naïve chronic hepatitis B patients treated with tenofovir (TDF) or entecavir (EVT). A. The incidence of malignant lymphoma in treatment-naïve CHB patients did not differ between those treated with TDF and those treated with EVT from 2012 to 2014. B. The incidence of malignant lymphoma in CHB patients without co-morbidities (DM, HTN, CKD, or LC) did not differ between those initially treated with TDF and those treated with ETV.

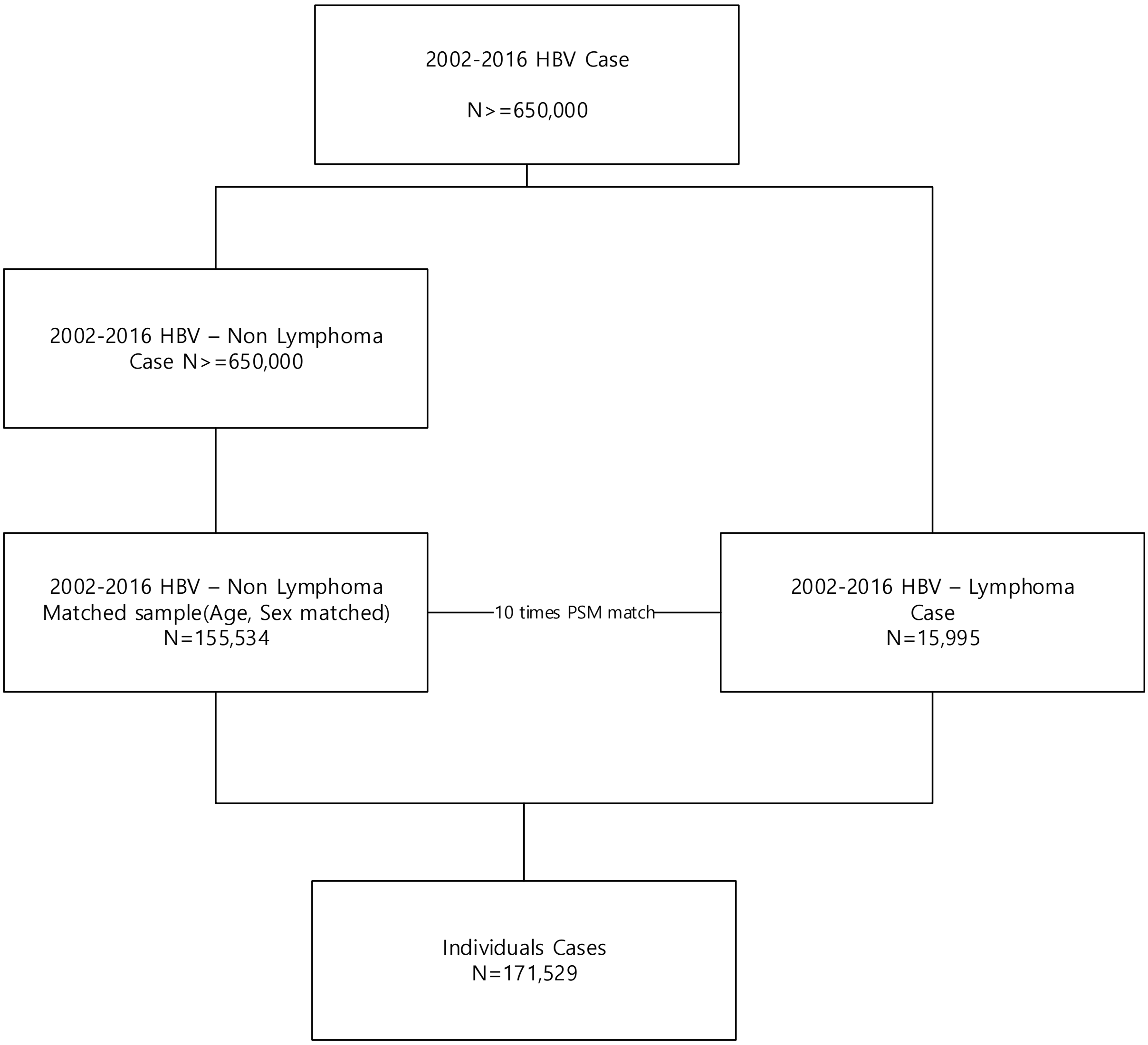

Supplement Figure S1. Study flow of chronic hepatitis B patients with and without malignant lymphoma from 2002 to 2016.

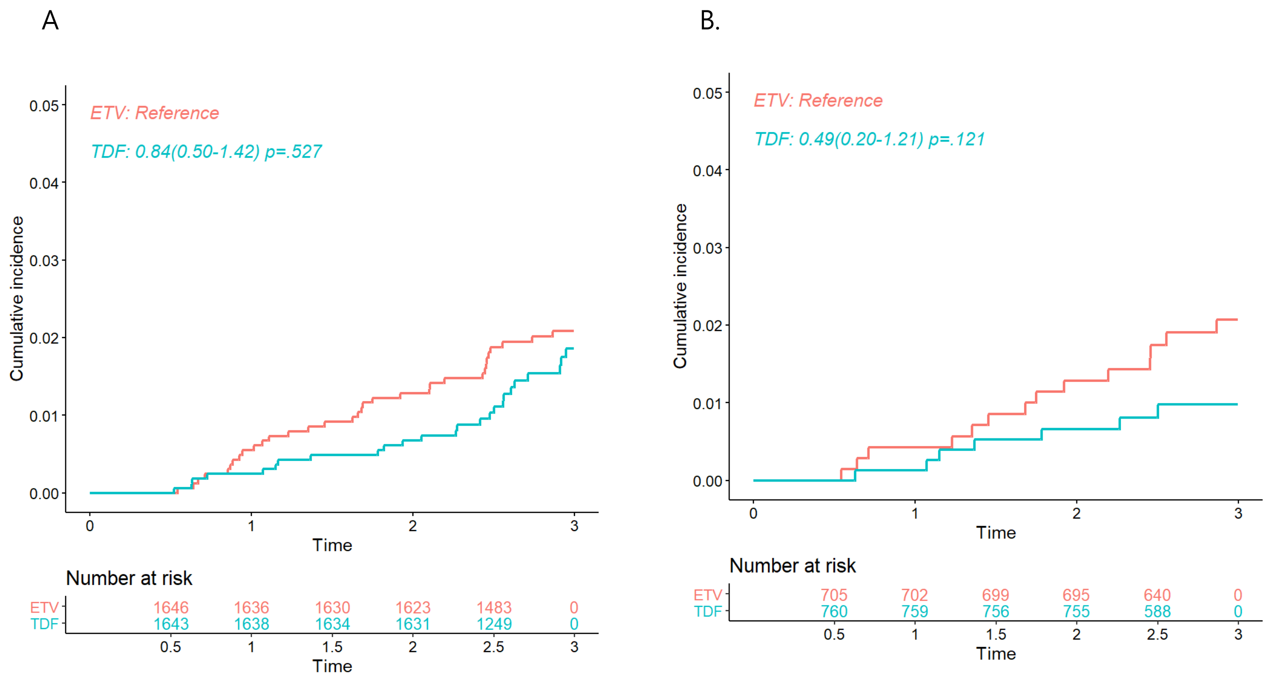

Supplement Figure S2. Newly diagnosed malignant lymphoma incidence in treatment-naïve chronic hepatitis B patients treated with tenofovir (TDF) or entecavir (EVT). A. The incidence of malignant lymphoma in treatment-naïve CHB patients did not differ between those treated with TDF and those treated with EVT from 2012 to 2014. B. The incidence of malignant lymphoma in CHB patients without co-morbidities (DM, HTN, CKD, or LC) did not differ between those initially treated with TDF and those treated with ETV.
