# Supplementary material for: Chronic Hepatitis B Viral Activity Enough to Take Antiviral Drug Could Predict the Survival Rate in Malignant Lymphoma
Source: Viruses. 2022 Aug 31;14(9):1943. doi: 10.3390/v14091943 (PMC9500776; doi:10.3390/v14091943)
Supplement: Supplementary file 1 [file viruses-14-01943-s001.zip › Viruses Supplement table.pdf]

Supplement data.

Supplement Table S1. Exclusion diagnosis codes of the National Health Insurance Service (NHIS).

Exclusion diagnosis	Codes
Hepatitis A	B15.0, B15.9
Hepatitis C	B17.1, B18.2
Hepatitis D	B18.0
Hepatitis E	B17.2
Acute hepatitis B	B16.0, B16.1, B16.2, B16.9, B17.0
Other viral hepatitis	B17.8, B17.9
AIDS	B20.0, B20.1, B20.2, B20.3, B20.4, B20.5, B20.6, B20.7, B20.8, B20.9<br>B21.0, B21.1, B21.2, B21.3, B21.7, B21.8, B21.9<br>B22.0, B22.1, B22.2, B22.7, B23.0, B23.1, B23.2, B23.8, B24.0
Transplantation	Z94.4, T86.4<br>V005, V013, V014, V015
Hepatocellular carcinoma	C22.0, C22.1, C22.2, C22.3, C22.4, C22.5

Supplement Table S2. Decompensation definition codes of the NHIS.

Decompensation	ATC codes	Korean Drug Codes
**Medication**		
Spironolactone	C03DA01	231101ATB, 231102ATB, 262700ATB
Terlipressin	H01BA04	236001BIJ, 236003BIJ, 236030BIJ
Somatostatin	B02BX	230201BIJ, 230203BIJ
Propranolol (with K74)	C07AA05	219901ATB, 219902BIJ, 219904ATB, 219905ACR, 219906ACR
**Procedure**		
Abdominal paracentesis	C8050	
Endoscopic treatment of esophageal or gastric varices	Q7631, Q7633	

Supplement Table S3. Co-morbidity diagnosis codes of the NHIS.

**Co-morbidity diagnosis**	**Codes**
Diabetes Mellitus	E10.00, E10.01, E10.02, E10.03, E10.08, E10.10, E10.11, E10.12, E10.18, E10.20, E10.21, E10.22, E10.28, E10.31, E10.32, E10.33, E10.34, E10.38, E10.40, E10.41, E10.42, E10.48, E10.50, E10.51, E10.58, E10.60, E10.61, E10.62, E10.63, E10.64, E10.68, E10.70, E10.71, E10.72, E10.78, E10.8, E10.9, E11.00, E11.01, E11.02, E11.03, E11.08, E11.10, E11.11, E11.12, E11.18, E11.20, E11.21, E11.22, E11.28, E11.31, E11.32, E11.33, E11.34, E11.38, E11.40, E11.41, E11.42, E11.48, E11.50, E11.51, E11.58, E11.60, E11.61, E11.62, E11.63, E11.64, E11.68, E11.70, E11.71, E11.72, E11.78, E11.8, E11.9 E14.00, E14.01, E14.02, E14.03, E14.08, E14.10, E14.11, E14.12, E14.18, E14.20 E14.21, E14.22, E14.28, E14.31, E14.32, E14.33, E14.34, E14.38, E14.40 E14.41, E14.42, E14.48, E14.50, E14.51, E14.58, E14.60, E14.61, E14.62, E14.63, E14.64, E14.68, E14.70, E14.71, E14.72, E14.78, E14.8, E14.9
Hypertension	I10.1, I10.9, I11.0, I11.9, I12.0, I12.9, I13.0, I13.1, I13.2, I13.9, I15.0, I15.1, I15.20, I15.21, I15.22, I15.28, I15.80, I15.88, I15.9
Chronic Kidney Disease	N18.1, N18.2, N18.3, N18.4, N18.5, N18.9

Supplement Table S4. Antiviral drug codes of the NHIS.

Antiviral drugs	ATC codes	Korean Drug Codes
Lamivudine	J05AF05, J05AR01, J05AR02, J05AR13	180901ASY, 180901ATB, 180902ATB, 1809030ASY, 513100ATB, 517300ATB
Adefovir	J05AF08	457501ATB
Clevudine	J05AF12	487801ACH, 487802ACH, 487803ACH
Telbivudine	J05AF11	506001ATB
Entecavir 0.5mg	J05AF10	487201ASY, 487202ATB, 487202ARD
Entecavir 1.0mg	J05AF10	487203ATB, 487203ATD, 487230ASY
Tenofovir	J05AR08, J05AF07, J05AR03, J05AR09	248100ATB, 493901ATB, 599900ATB, 623400ATB

Supplement Table S5. Cumulative data for all HBV-related malignant lymphoma patients in Korea.

year	HBV Cases	MBCL	TNK	Hodgkin	Unknown	Total
2002	295,856	648	46	42	7	743
2003	320,182	984	64	66	10	1124
2004	330,524	1279	82	88	9	1458
2005	371,285	1653	103	103	12	1871
2006	387,894	1985	134	112	14	2245
2007	426,011	2312	161	119	15	2607
2008	446,601	2681	181	136	19	3017
2009	473,259	3125	214	153	18	3510
2010	482,848	3559	249	182	22	4012
2011	522,507	4151	287	226	22	4686
2012	512,352	4712	323	268	28	5331
2013	516,650	5344	381	295	29	6049
2014	536,837	5961	436	320	34	6751
2015	576,002	6798	532	366	41	7737
2016	646,273	7942	653	398	49	9042

MBCL, Mature B cell lymphoma; TNK, Mature T cell and NK-cell lymphoma; Hodgkin, Hodgkin lymphoma; Unknown, Unknown type of lymphoid neoplasm

Supplement Table S6. Mean and median age of newly diagnosed malignant lymphoma patients with chronic hepatitis B virus in Korea.

year	Mean	SD	Q2 (median)	IQR
2003	51.10	15.25	52	(41-62)
2004	52.18	14.60	53	(43-63)
2005	51.11	15.31	53	(41-62.5)
2006	52.87	14.86	54	(44-64)
2007	53.59	14.66	54	(45-65)
2008	53.12	14.84	54	(44-64)
2009	53.29	15.47	54	(43-65)
2010	53.45	14.70	54	(45-64)
2011	53.96	15.00	55	(44-65)
2012	53.36	15.31	53	(43-65)
2013	53.48	15.35	55	(44-65)
2014	53.76	15.09	54	(44-65)
2015	54.43	15.56	55	(44-66)
2016	55.06	15.23	55	(46-66)

Supplement Table S7. Prognostic factors of malignant lymphoma with chronic hepatitis B virus.

	Univariate		Multivariate	
	HR(95%CI)	p-value	HR(95%CI)	p-value
Chronic hepatitis B				
Age	1.04(1.03-1.04)	<.001	1.04(1.03-1.04)	<.001
Sex-male				
female	0.74(0.70-0.79)	<.001	0.74(0.70-0.79)	<.001
Diabetes mellitus	1.55(1.46-1.65)	<.001	1.17(1.09-1.25)	<.001
Hypertension	1.54(1.44-1.64)	<.001	1.06(0.98-1.14)	0.136
Cirrhosis	1.78(1.60-1.97)	<.001	1.64(1.47-1.83)	<.001
Antivirals	1.20(1.07-1.34)	0.002	1.28(1.13-1.45)	<.001
Liver cirrhosis				
Age	1.04(1.03-1.06)	<.001	1.04(1.03-1.06)	<.001
Sex-male				
female	1.03(0.74-1.42)	0.874	0.92(0.66-1.29)	0.64
Diabetes mellitus	1.02(0.76-1.37)	0.885	0.98(0.72-1.33)	0.887
Hypertension	1.16(0.86-1.57)	0.338	0.9(0.67-1.29)	0.66
Antivirals	0.74(0.53-1.02)	0.069	0.67(0.48-0.94)	0.022
